# Clinical utility of ANA-ELISA vs ANA-immunofluorescence in connective tissue diseases

**DOI:** 10.1038/s41598-021-87366-w

**Published:** 2021-04-15

**Authors:** Omar Suhail Alsaed, Laith Ishaq Alamlih, Omar Al-Radideh, Prem Chandra, Samar Alemadi, Abdul-Wahab Al-Allaf

**Affiliations:** Department of Medicine, Division of Rheumatology, Hamad Medical Corporation, P. O. BOX 3050, Doha, Qatar

**Keywords:** Biomarkers, Rheumatology

## Abstract

We investigated the performance of ANA-ELISA for CTDs screening and diagnosis and comparing it to the conventional ANA-IIF. ANA-ELISA is a solid-phase immune assay includes 17 ANA-targeted recombinant antigens; dsDNA, Sm-D, Rib-P, PCNA, U1-RNP (70, A, C), SS-A/Ro (52 and 60), SS-B/La, Centromere B, Scl-70, Fibrillarin, RNA Polymerase III, Jo-1, Mi-2, and PM-Scl. During the period between March till December 2016 all requests for ANA from primary, secondary, and tertiary care centers were processed with both techniques; ANA-IIF and ANA-ELISA. The electronic medical record of these patients was reviewed looking for CTD diagnosis documented by the Senior rheumatologist. SPSS 22 is used for analysis. Between March and December 2016, a total of 12,439 ANA tests were requested. 1457 patients were assessed by the rheumatologist and included in the analysis. At a cut-off ratio ≥ 1.0 for ANA-ELISA and a dilutional titre ≥ 1:80 for ANA-IIF, the sensitivity of ANA-IIF and ANA-ELISA for all CTDs were 63.3% vs 74.8% respectively. For the SLE it was 64.3% vs 76.9%, Sjogren’s Syndrome was 50% vs 76.9% respectively. The overall specificity of ANA-ELISA was 89.05%, which was slightly better than ANA-IIF 86.72%. The clinical performance of ANA-ELISA for CTDs screening showed better sensitivity and specificity as compared to the conventional ANA-IIF in our cohort.

## Introduction

Antinuclear antibody detection by indirect immunofluorescence technique (ANA-IIF) is a valuable screening tool for autoimmune connective tissue diseases (CTDs), though it is non-specific. The test can be positive in many autoimmune conditions other than CTDs such as autoimmune hepatitis, primary biliary cirrhosis, Hashimoto thyroiditis Etc. It can be false positive as well in other circumstances such as non-autoimmune diseases like cancers, infections, in patients taking certain medications like antiepileptics and antiarrhythmics and in asymptomatic first-degree relatives of patients with autoimmune diseases^1^.

The two main methods to detect ANA are the indirect immunofluorescence ANA-IIF and the ELISA technique. ANA-IIF is the current endorsed technique for ANA detection by the American and Europeans rheumatology Societies (ACR and EULAR). It has poor specificity and low positive predictive value especially when low titers are used as a cutoff. At a titre of 1:40 serum dilution, 25–30% of healthy individuals might test positive for ANA, which increases with age^2,3^. Overall, it is recommended that the serum dilution that gives a specificity of 95% in healthy individuals should be used as the cut-off^4^. It was found that the clinical significance of the test rises with increasing titers^5,6^, as well as with the identification of the responsible specific autoantigen^7^. Despite the good sensitivity of the test, ANA-IIF has some limitations; it is a time-consuming, labor-intensive and operator dependent test. Determining the correct dilutional titer depends on the experience of the technician who is reading the immunofluorescence slides.

For the last two decades, ANA testing with ELISA technique has been introduced aiming to save time and efforts needed for ANA-IIF and trying to improve the performance of the ANA testing. However, previous reports showed that solid-phase assays still have lower sensitivity when compared to indirect immunofluorescence^8^.

Manufacturers of solid-phase assays attempt to improve the performance of the assays by adding extra purified recombinant antigens. Recently a new ANA-ELISA was introduced by Phadia company for connective tissue disease screen that includes 17 different antigens (dsDNA, SSA/Ro (52+ 60), SSB/La, U1-RNP (RNP-70, A, C), Sm, centromere B, Jo-1, Scl-70, Rib-P, fibrillarin, RNA Pol III, PM-Scl, PCNA and Mi-2)^9^. Up to date, there is a limited data about the clinical utility, sensitivity, specificity and the clinically significant ratio of this assay. Accordingly, the main objective of this study is to test the clinical utility of the new ANA-ELISA for CTDs diagnosis in comparison to ANA-IIF by calculating the sensitivity, specificity, positive predictive value (PPV) and negative predictive value (NPV).

## Materials and methods

The study has been conducted at Hamad Medical Corporation (HMC) in Qatar. HMC immunology lab is a central lab that processes all ANA samples requested from primary and secondary health care physicians. The new ANA-ELISA was introduced to HMC on 1st of March 2016. For validation purpose, all blood samples requested for ANA during the period 1st of March to 31st of December 2016 were processed for both techniques. We, then retrospectively reviewed all medical records of the patients who were evaluated by senior rheumatologists irrespective of their test results.

### Anti-nuclear antibody testing

ANA-ELISA was performed using (ELISA) kits (Phadia GmbH, Freiburg, Germany) performed on the Phadia-250 automated platform. The ANA-ELISA assay contains 17 ANA-targeted recombinant antigens including; dsDNA, Sm-D, Rib-P, PCNA, U1-RNP (70, A, C), SS-A/Ro, SS-B/La, Centromere B, Scl-70, Fibrillarin, RNA Polymerase III, Jo-1, Mi-2, and PM-Scl. The test results are expressed as a ratio, which is positive if ≥1.0, equivocal if 0.7-0.99 and negative if <0.7. The ANA-IIF was performed using (Diasorin S.P.A, Via Crescentino snc, 13040 Saluggia VC, Italy). The cut off for positive results was 1:80 or higher (4). Further testing for dsDNA and other extractable nuclear antigens (ENA) was undertaken on a subset of sera that were positive for ANA-IIF or whenever there was a discrepancy between the two methods of ANA detection.

Both ANA testing methods were run according to the standard regulations and quality. The central immunology lab of HMC is accredited by College of American Pathologist (CAP) and it is under regular quality control check.

### Electronic records screening and CTD diagnosis

Two independent reviewers (rheumatologists) screened all patients who were assessed in rheumatology clinics within two years of ANA testing (one year before and one year after). The reviewers then evaluated the electronic records of those patients, looking for CTD diagnosis documented by the rheumatologists (expert in the field). The presence or the absence of a specific diagnosis of CTD was based on the evaluation of the rheumatologist as documented in the electronic records.

### Statistical analysis

Descriptive statistics were used to summarize and determine the sample characteristics and distribution of responses. The normally distributed data and results were reported with mean and standard deviation (SD); the remaining results were reported with median and interquartile range (IQR). Categorical data were summarized using frequencies and percentages.

The primary aim of this study is to determine and compare the clinical utility of ANA-IIF and ANA-ELISA in CTD screening. Various diagnostic accuracy and performance measures such as sensitivity, specificity, positive predictive value (PPV), and negative predictive value (NPV) were computed and presented with corresponding 95% confidence limits to measure the precision of the estimated values.

ANA results were classified as positive or negative for each patient. Positive predictive values for a "positive ANA test result" were further evaluated after stratification according to the titer, as well as according to clinically relevant and crucial significant patient characteristics. Positive and negative likelihood ratios (LRs) were calculated and interval LRs, defined as the probability of obtaining a test result in a specific range when a CTD is presently divided by the probability of obtaining that test result when a CTD is absent, it was calculated to capture the magnitude of abnormality of the test results.

All Statistical analyses were done using statistical packages SPSS 22 (SPSS Inc. Chicago, IL) and Epi Info 2000 (Centers for Disease Control and Prevention, Atlanta, GA).

### Ethical approval

All methods were carried out under the relevant guidelines and regulations. This study was granted by the medical research center of Hamad Medical Corporation under protocol number 01–17-013. Waiver consent was used to review subject’s electronic medical records by the Internal Review Board (IBR) committee at the Hamad Medical Institute (HMC).

## Results

**Figure 1:**
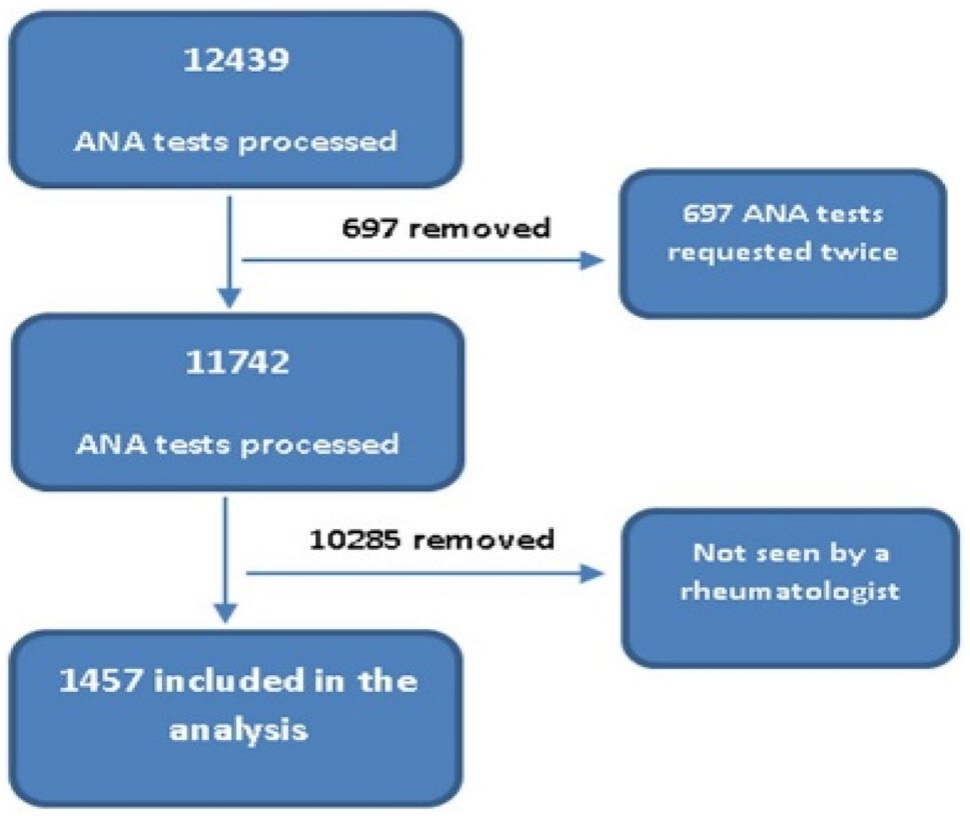
Scheme of study subjects.

Between March and December 2016, 12439 ANA tests were requested from all HMC and PHC hospitals (primary, secondary and tertiary health care). There were 697 duplicated tests. 1457 out of the remaining 11742 (12.4%) were evaluated in rheumatology clinics for autoimmune connective tissue diseases. (Figure 1).

1457 subjects’ sera were included in the study. 75.7% were females with a mean age of 43±13 years. Majority of our patients were Arabs (61.9%), followed by Asian (32.6%) and others (5.5%). ANA-IIF was positive in 293 (20.1%) at a dilution titer of ≥1:80 and 197 (13.5%) at a dilutional titer ≥1:160. ANA-ELISA was positive in 288 (19.7%) at a ratio ≥1 and 215 (14.7%) at a ratio ≥2. The diagnosis of CTD was confirmed clinically in 199 (13.6%) patients out all the cohort of 1457 patients. The most prevalent diagnosis was SLE in 143/199 (71.8%), followed by Sjogren's syndrome in 26/199 (13%), then scleroderma in 15/199 (7.5%), inflammatory myositis in 9/199 (4.5%), undifferentiated CTD in 10/199 (5%) and mixed connective disease 3/199 (1.5%). Seven patients were diagnosed with two connective tissue diseases like SLE with secondary Sjogren's disease.

### The clinical performance of ANA-IIF alone, ANA-ELISA alone and a combination of both techniques

For all those with positive ANA-IIF ≥1:80 and ≥ 1:160, regardless of their ANA-ELISA status; 126/293 (43%) and 104/197 (52.7%) diagnosed with CTD with sensitivity of 63.3% (95% CI 56.2% to 70.0%) and 52.2% (95% CI 45.08% to 59.37%), respectively. For those with a ratio of ≥1 for ANA-ELISA regardless of their ANA-IIF status; 149/288 (51%) diagnosed with CTD with the sensitivity of 74.8% (95% CI 68.2% to 80.7%). Combining both tests (either test positive) improved the sensitivity to 82.4% (95% CI 76.40% to 87.43%).

For those with negative ANA-IIF (ANA-IIF $\le 1:80$), regardless of their ANA-ELISA status; only 73/1164 (6.2%) diagnosed with CTD with negative predictive value 93.7%. On the other hand, those with negative ANA-ELISA ratio of < 1) regardless of their ANA-IIF status, only 50/1180 (4.2%) diagnosed with CTD with negative predictive value 95.7%. Combining both tests (both tested negative), improves slightly the negative predictive value of 96.6%. Table.1 (see supplement for detailed calculation with 95% CI).

**Table 1:** Clinical performance of both ANA techniques in CTD diagnosis.

Statistic	ANA-ELISA ≥ 1	ANA-IIF ≥ 1:80	ANA-IIF ≥ 1:160	ANA-IIF ≥ 1:80 and/or ANA-ELISA ≥ 1
Sensitivity	74.87%	63.32%	52.26%	82.41%
Specificity	89.05%	86.72%	92.61%	78.93%
Positive Predictive Value	51.74%	43.00%	52.79%	38.23%
Negative Predictive Value	95.76%	93.73%	92.46%	96.60%
Accuracy	87.13%	83.53%	87.10%	79.41%
Positive Likelihood Ratio	6.84	4.77	7.07	3.91
Negative Likelihood Ratio	0.28	0.42	0.52	0.22

For those who were negative for ANA-IIF and positive for ANA-ELISA, 26/199 (13%) were clinically confirmed to have CTD by rheumatologist assessment; 18 SLE, 6 Sjogren’s syndrome, 2 inflammatory myositides, 1 mixed connective tissue disease, 1 undifferentiated connective tissue disease. While, those who were positive for ANA-IIF and negative for ANA-ELISA, 9/199 (4.5%) were diagnosed clinically with CTD by rheumatologist assessment; 6 SLE and three inflammatory myositides.

### Disease-dependent performance of ANA-IIF and ANA-ELISA

In general, the clinical performance of ANA-ELISA was higher than conventional ANA-IIF in various CTDs (Table 2). The sensitivity of ANA-ELISA at a ratio ≥1 was greater than the ANA-IIF at a titre of ≥1:80 in all CTDs except for systemic sclerosis (Table 3).

**Table 2:** Relationship of various CTD and different ANA detection techniques at different dilutional titers and ratios.

Diagnosis N (%)	CTD 199 (100)	SLE 143 (100)	Ss 26 (100)	Scl 15 (100)	IM 9 (100)	MCT 3 (100)	UCTD 10 (100)
ANA-IIF ^negative^	50 (25)	34 (24)	11(42)	1 (7)	4 (44)	1 (30)	1 (10)
ANA-IIF 1:40	23 (12)	17 (12)	2 (8)	2 (13)	1 (11)	0	3 (30)
ANA-IIF 1:80	126 (63)	92 (64)	13 (50)	12 (80)	4 (44)	2 (67)	6 (60)
ANA-IIF 1:160	104 (52)	78 (55)	9 (35)	10 (67)	3 (33)	2 (67)	5 (50)
ANA-ELISA ^negative^	39 (20)	26 (18)	4 (15)	2 (13)	6 (67)	0	1 (10)
ANA-ELISA ^equivocal^	11 (6)	7 (5)	2 (8)	2 (13)	0	0	0
ANA-ELISA ≥ 1	149 (75)	110 (77)	20 (77)	11 (73)	3 (33)	3 (100)	9 (90)
ANA-ELISA ≥ 2	136 (68)	101 (71)	20 (77)	10 (67)	3 (33)	1 (30)	8 (80)
ANA-IIF 1:80 and/or ANA-ELISA ≥ 1	111(55.8)	120 (83)	20 (76)	13 (86)	6 (66.6)	3 (100)	9 (90)
Both negative; ANA-ELISA & ANA-IIF	19 (9.5)	13 (9)	3 (12)	1 (7)	2 (22)	0	0

**CTD**: Connective Tissue Disease, **SLE**: Systemic Lupus Erythematosus, **SS**: Sjogren’s syndrome, **Scl**: scleroderma, **IM**: inflammatory myositis, **MCTD**: Mixed Connective Tissue Disease, **UCTD**: Undifferentiated Connective Tissue Disease. Seven patients were having SLE with secondary SS at the same time.

**Table 3:** Disease-dependent sensitivity for ANA-IIF at a dilutional titer of 1:80 and ANA-ELISA at ratio > 1.

	ANA-IIF ≥ 1:180	ANA-ELISA
CTD	63.3%	76.9%
SLE	64.3%	76.9%
Ss	50%	76.9%
Scl	80%	73.3%
IM	44%	66.6%
MCTD	66.6%	100%
UCTD	60%	90%

CTD: Connective Tissue Disease, SLE: Systemic Lupus Erythematosus, Ss: Sjogren’s syndrome, Scl: scleroderma, IM: inflammatory myositis, MCTD: Mixed Connective Tissue Disease, UCTD: Undifferentiated Connective Tissue Disease.

### The sensitivity of different ANA techniques to detect various extractable nuclear antigens (ENAs)

Generally, the performance of ANA-ELISA in ENAs detection was better than ANA-IIF. The sensitivity of ANA-ELISA in the detection of anti-Ds DNA, anti-Ro and anti-La (95.8%, 98.6 and 100%) was higher comparing to ANA-IIF (73.6%, 61.8% and 83.3%), respectively. Table 4 compares the performance of ANA-IIF and ANA-ELISA in the detection of different extractable nuclear antigens (ENAs).

**Table 4:** Comparison of performance of ANA-IIF and ANA-ELISA in the detection of different extractable nuclear antigens.

	Anti-Ds DNA	Anti-SM	Anti-Ro	Anti-La	Anti-Rib-p	Anti-Scl	Anti-RNP	Anti-Jo	Anti-CENP
ANA-IIF ^negative^	11	3	20	3	1	1	0	2	0
ANA-IIF 1:40	8	1	9	1	0	0	0	0	1
ANA-IIF 1:80	53	13	47	20	6	5	23	0	8
Sensitivity %	73.6	76.4	61.8	83.3	85.7	83.3	100		88.9
ANA-ELISA ^negative^	1	0	0	0	1	0	0	0	0
ANA-ELISA ^equivocal^	2	2	1	0	0	0	2	0	0
ANA-ELISA > 1	69	15	75	24	6	6	21	2	9
Sensitivity %	95.8	88.2	98.6	100	85.7	100	91.3	100	100

### Concordance and discordance between ANA-IIF and ANA-ELISA

Ninety-one (6.2%) serum samples were positive for ANA-ELISA and negative for ANA-IIF and 118 (8%) serum samples were positive for ANA-IIF and negative for ANA-ELISA. At a dilution titer of ≥1:80 for ANA-IIF and at a ratio of ≥1 for ANA-ELISA, the concordance for positive ANA-IIF and ANA-ELISA was 152 (10.4% were positive for both) and for negative ANA-IIF and ANA-ELISA was 869 (59.6% were negative for both). The overall concordance between both techniques is 70.0% [(869+152) /1457 * 100]. Table 5 demonstrates the relationship between ANA-IIF and ANA-ELISA at a different cut off points.

**Table 5:** Relationship between ANA-IIF and ANA-ELISA at the different cut off points.

	ANA-ELISA ^negative^	ANA-ELISA ^equivocal^	ANA-ELISA > 1	Total
ANA-IIF ^negative^	869	32	91	992
ANA-IIF 1:40	113	14	45	172
ANA-IIF 1:80	118	23	152	293
Total	1100	69	288	1457

## Discussion

Proper interpretation of ANA-IIF and ANA-ELISA in CTD screening is critical for clinicians who are dealing with different ANA-associated autoimmune diseases. ANA-IIF sensitivity for CTD depends on the specific type of connective tissue disease screened for. This mainly depends on the number and type of autoantibodies involved in that disease. For example; ANA-IIF sensitivity for SLE and scleroderma is 93% and 84%, respectively, while for other CTDs, it ranges from 40% to 64% which is considered low for the clinical use^10^. There are many ENAs involved in SLE; ds-DNA, anti-Ro, anti-RNP, anti-Rib p and anti-Sm. While other connective tissue diseases have one or two known ENAs e.g; Sjogren’s disease and scleroderma.

The performance of ANA-ELISA in our cohorts for detecting ds-DNA and anti-Ro was 95% and 100%, while ANA-IIF detected 73% and 61%, respectively (Table 4). It is well known that Anti-Ro and Anti ds-DNA can be missed by ANA-IIF technique^9,11,12^. Another reason for having low ANA-IIF sensitivity in non-SLE CTD e.g: polymyositis/dermatomyositis (PM/DM) is that, these conditions have anti-cytoplasmic antibodies which target extra-nuclear antigens that are not reported by ANA-IIF as a routine. For that, the presence of the anti-cytoplasmic antibodies in the immune assay of ELISA may have an advantage in improving the performance ANA-ELISA over ANA-IIF in PM/DM.

In our cohort, the sensitivity of ANA-IIF for CTDs, in general, was 63.3% and for SLE specifically, 64.3% which is low compared to the global figures^3,9^, however, there is a recent study with similar methodology which showed low sensitivity for ANA-IIF^13^. This could be because most of the ANA tests were requested from secondary care physicians and non-rheumatology specialties. Usually, these requests were done out of the clinical context. Also, it is well known that the ANA seroconversion can happen later on and in some even after receiving treatment.

There are a few proposed algorithms for which ANA technique should be used in CTD screening. It depends on laboratory standardization for the immunofluorescence technique and microscopist experience and the number of the purified recombinant antigens incorporated in the immune assay. Another suggestion is that the choice of ANA technique could be disease specific. In centers where the local performance of ANA-IIF is not up to the global figures and with the large scale of out of clinical context ANA requests for CTD screening, as in our center, it might be advisable to start screening with ANA-ELISA rather than ANA-IIF. This is because the ELISA is standardized and automated and it is not manual intensive or technicians dependent as compared to ANA-IIF. A recent meta-analysis concluded that the double-testing strategy using IIF and ELISA has more clinical value than IIF or ELISA alone^14^.

Clinicians should be oriented for the cellular and molecular characteristics of the new autoantibodies and the performance of each ANA technique on these new autoantibodies. For example, there are more than 20 known autoantibodies involved in the pathophysiology of autoimmune inflammatory polymyositis. Some of them target nuclear antigens e.g; PM-Scl, Mi2 and anti-Ku^15–17^ and others target cytoplasmic antigens e.g; anti-Jo, MDA-5 and SRP^18–20^. The solid-phase assay of ELISA used in our study contains 3 of PM/DM autoantibodies only (Mi2, anti-Jo and Pm-Scl), so it is not a good screening tool for inflammatory myositis. On the contrary, most of the systemic sclerosis autoantibodies (Scl-70, RNA polymerase III, anti-centromere, anti-fibrillarin, Pm-Scl and U3-RNP) are incorporated in the used solid-phase assay of the ELISA, which increased the yield of the test for systemic sclerosis screening.

Our study highlighted the performance of ANA-ELISA on a large number of patients. It showed generally comparable results to ANA-IIF, which is the gold standard for ANA detection. In a recent metanalysis, the sensitivity of ANA-IIF was better than ANA-ELISA. On the other hand, the specificity of ANA-ELISA was higher the ANA-IIF. Different immune assays were used for ANA-ELISA in the studies that were included in the meta-analysis. Also, the methodology of the included studies was heterogeneous^14^. Finally, due to rarity of some of the CTDs such as inflammatory myositis, undifferentiated CTDs and mixed CTDs makes it difficult to have conclusions regarding the performance of the test in these diseases. Also, due to the retrospective nature of the study which increases the risk of bias, the results should be taken with caution. More prospective studies are needed on a larger scale to confirm these findings.

We have to admit that establishing a diagnosis of a CTD from case review can be challenging, as CTD can evolves over time. We did our best to collect as much information as we can and all as documented by senior consultants rheumatologist who looks after these cases. Another point for discussion is related to the fact that we excluded the 10285 as they have not been assessed by rheumatologist and we can not rely on non-rheumatologist notes re that. However, we want to point that the whole sample of the 10285 and the selected 1457 has drawn from the same sample for blood tests ordered by different physicians in primary and secondary settings.

The ANA-ELISA also has the disadvantage of not including the pattern of ANA which could have some relevant information to the treating physician with regards to the possible diagnosis. However, we can rely more on the clinical scenario in making our diagnosis. We also do not know how other non-CTD conditions could affect the results of the test, including the false positive or negatives. The new test is also not comprehensive and lacks some of other auto-antigens such as MAD-5.

In conclusion, we have evaluated the diagnostic utility of the most recently developed solid-phase screening assay (CTD-Screen) for the detection of ANA in a "real-life setting". We found the new ANA-ELISA has comparable diagnostic accuracy to the commonly used ANA-IIF in CTD. However, ANA-ELISA has the advantage of being less manual, not operating dependent and is more sensitive and specific than the “gold standard” indirect immunofluorescence (ANA-IIF).

## Supplementary Information

Supplementary Information

## Data Availability

All data stored on a secured computer system and available.
