# Supplementary material for: Clinical utility of ANA-ELISA vs ANA-immunofluorescence in connective tissue diseases
Source: Sci Rep. 2021 Apr 15;11:8229. doi: 10.1038/s41598-021-87366-w (PMC8050204; doi:10.1038/s41598-021-87366-w)
Supplement: Supplementary file 1 — Supplementary Information [file 41598_2021_87366_MOESM1_ESM.docx]

**Calculations of the clinical performance of ANA-ELISA ≥1**

|  | **CTD positive** | **CTD negative** |
| --- | --- | --- |
| **ANA-ELISA positive** | 149 | 139 |
| **ANA-ELISA negative** | 50 (including equivocal ANA-ELISA) | 1130 |

| **Statistic** | **Value** | **95% CI** |
| --- | --- | --- |
| **Sensitivity** | 74.87% | 68.25% to 80.74% |
| **Specificity** | 89.05% | 87.20% to 90.71% |
| **Positive Predictive Value** | 51.74% | 47.33% to 56.11% |
| **Negative Predictive Value** | 95.76% | 94.67% to 96.64% |
| **Accuracy** | 87.13% | 85.30% to 88.80% |
| **Positive Likelihood Ratio** | 6.84 | 5.73 to 8.15 |
| **Negative Likelihood Ratio** | 0.28 | 0.22 to 0.36 |

**Calculations of the clinical performance of ANA-IIF ≥1:80**

|  | **CTD positive** | **CTD negative** |
| --- | --- | --- |
| **ANA-IIF positive** | 126 | 167 |
| **ANA-IIF negative** | 73 (including ANA-IIF 1:40) | 1091 |

| **Statistic** | **Value** | **95% CI** |
| --- | --- | --- |
| **Sensitivity** | 63.32% | 56.21% to 70.02% |
| **Specificity** | 86.72% | 84.72% to 88.55% |
| **Positive Predictive Value** | 43.00% | 38.74% to 47.37% |
| **Negative Predictive Value** | 93.73% | 92.56% to 94.73% |
| **Accuracy** | 83.53% | 81.52% to 85.40% |
| **Positive Likelihood Ratio** | 4.77 | 4.00 to 5.69 |
| **Negative Likelihood Ratio** | 0.42 | 0.35 to 0.51 |

**Calculations of the clinical performance of ANA-IIF ≥1:160**

|  | **CTD positive** | **CTD negative** |
| --- | --- | --- |
| **ANA-IIF positive** | 104 | 93 |
| **ANA-IIF negative** | 95 (including ANA-IIF 1:40) | 1165 |

| **Statistic** | **Value** | **95% CI** |
| --- | --- | --- |
| **Sensitivity** | 52.26% | 45.08% to 59.37% |
| **Specificity** | 92.61% | 91.02% to 93.99% |
| **Positive Predictive Value** | 52.79% | 46.89% to 58.62% |
| **Negative Predictive Value** | 92.46% | 91.38% to 93.42% |
| **Accuracy** | 87.10% | 85.27% to 88.78% |
| **Positive Likelihood Ratio** | 7.07 | 5.58 to 8.95 |
| **Negative Likelihood Ratio** | 0.52 | 0.45 to 0.60 |

**Calculations of the clinical performance of combining ANA-IIF ≥1:80 and/or ANA-ELISA ≥1**

**(Either test positive)**

|  | **CTD positive** | **CTD Negative** |
| --- | --- | --- |
| **Either test positive**  **ANA-IIF & ANA-ELISA** | 164 | 265 |
| **ANA-IIF & ANA-ELISA negative** | 35 (including ANA-IIF 1:40 and including equivocal ANA-ELISA) | 993 |

| **Statistic** | **Value** | **95% CI** |
| --- | --- | --- |
| **Sensitivity** | 82.41% | 76.40% to 87.43% |
| **Specificity** | 78.93% | 76.58% to 81.16% |
| **Positive Predictive Value** | 38.23% | 35.33% to 41.21% |
| **Negative Predictive Value** | 96.60% | 95.45% to 97.46% |
| **Accuracy** | 79.41% | 77.24% to 81.46% |
| **Positive Likelihood Ratio** | 3.91 | 3.45 to 4.43 |
| **Negative Likelihood Ratio** | 0.22 | 0.16 to 0.30 |
